# Interaction between *ELMO1* gene polymorphisms and environment factors on susceptibility to diabetic nephropathy in Chinese Han population

**DOI:** 10.1186/s13098-019-0492-0

**Published:** 2019-11-27

**Authors:** Yi Hou, Yong Gao, Yan Zhang, Si-Tong Lin, Yue Yu, Liu Yang

**Affiliations:** 1 Department of Urology, China-Japan Union Hospital of Jilin University, Changchun, 130033 Jilin People’s Republic of China; 2 Department of Critical Care, China-Japan Union Hospital of Jilin University, Changchun, 130033 Jilin People’s Republic of China; 3 Department of Endocrinology, China-Japan Union Hospital of Jilin University, Changchun, 130033 Jilin People’s Republic of China

**Keywords:** Diabetic nephropathy, Single nucleotide polymorphisms, Engulfment and Cell Motility 1, Interaction, Smoking, Hypertension, Alcohol

## Abstract

**Background:**

The association of diabetic nephropathy (DN) risk with single nucleotide polymorphisms (SNPs) within Engulfment and Cell Motility 1 (*ELMO1*) gene and gene–environment synergistic effect have not been extensively examined in, therefore, the purpose of this study is to explore the association between multiple SNPs in *ELMO1* gene, and the relationship between gene–environment synergy effect and the risk of DN.

**Methods:**

Genotyping for 4 SNPs was performed with polymerase chain reaction (PCR) and following restriction fragment length polymorphism (RFLP) methods. Hardy–Weinberg balance of the control group was tested by SNPstats (online software: http://bioinfo.iconologia.net/snpstats). The best combination of four SNPs of *ELMO1* gene and environmental factors was screened by GMDR model. Logistic regression was used to calculating the OR values between different genotypes of *ELMO1* gene and DN.

**Results:**

The rs741301-G allele and the rs10255208-GG genotype were associated with an increased risk of DN risk, adjusted ORs (95% CI) were 1.75 (1.19–2.28) and 1.41 (1.06–1.92), respectively, both *p*-values were < 0.001. We also found that the others SNPs-rs1345365 and rs7782979 were not significantly associated with susceptibility to DN. GMDR model found a significant gene–alcohol drinking interaction combination (*p *= 0.0107), but no significant gene–hypertension interaction combinations. Alcohol drinkers with rs741301-AG/GG genotype also have the highest DN risk, compared to never drinkers with rs741301-AA genotype, OR (95% CI) 3.52 (1.93–4.98).

**Conclusions:**

The rs741301-G allele and the rs10255208-GG genotype, gene–environment interaction between rs741301 and alcohol drinking were all associated with increased DN risk.

## Background

Diabetic nephropathy (DN) is one of the most destructive microvascular complications of all the complications of diabetes, and it is the main cause of end-stage renal disease (ESRD), which accounts for a large proportion of the morbidity and mortality of diabetes [1]. DN is also an important risk factor for the development of cardiovascular disease and chronic kidney disease [2] and one of the main causes of death [3]. The pathogenesis of DN was multifactorial and was also not been well known. Previous studies have reported that some genetic and environmental risk factors may lead to its progress, including hyperglycemia, hypertension and hyperlipidemia, which were common modifiable risk factors. In addition, some unmodifiable risk factors should also draw public attentions, such as genetic factors [4, 5].

Genome-Wide Association Studies (GWAS) and meta-analysis [6, 7] have reported some genes related with susceptibility to DN in different populations. In 2005, a Japanese GWAS study [8] confirmed that Engulfment and Cell Motility 1 (*ELMO1*) gene is a new candidate gene. In this study, they evaluated more than 80,000 loci and found that one SNP locus in intron 18 of *ELMO1* gene was closely related to DN. Then, some studies also reported that this gene was associated with kidney disease attributed to T2DM in different populations [9–11], including Chinese populations [11]. The detailed mechanism for association between *ELMO1* gene polymorphisms and DN susceptibility was not well known. However, a previous study [12] showed that mutations in elmo1 gene can lead to the disorder of extracellular matrix (ECM) metabolism, which leads to the accumulation of ECM, thickening of renal tubules and glomerular basement membrane, thus increasing the risk of DN. However, to date, the association between DN risk and SNPs within *ELMO1* gene have not been extensively examined in Chinese populations. Strong evidences [13, 14] have suggested the important role for the combination of environmental exposures and genetic factors for development or progression of the DN. However, till now, no study investigated the *ELMO1* gene–environment interaction. Therefore, the present study aimed to evaluate the impact of four SNPs in the *ELMO1* gene, and its interaction with environmental risk factors on susceptibility to DN.

## Materials and methods

### Subjects

There were a total of 1325 participants including 660 T2DM patients with DN and 665 T2DM patients without DN. Those participants with a fasting glucose ≥ 126 mg/dl (7.0 mmol/l), or a 2 h postprandial blood glucose ≥ 200 mg/dl (11.0 mmol/l), or if hypoglycemic therapy (oral agents or insulin) had been started were considered as T2DM patients [15]. The diagnostic criteria of DN was made according to World Health Organization 1999 [16]. Those T2DM patients were considered as cases with DN, including those with persistent urine albuminuria more than 300 mg/l in consecutive twice measurements, or without renal failure: serum creatinine more than 1 mg/dl. Those T2DM patients without DN were included in the control group. All subjects in the study received detailed clinical and biochemical examinations. Those T2DM patients with end-stage renal disease (ESRD) were excluded from both case and control group. All the subjects were Han people, and there was no genetic and blood relationship between any two subjects. Before participating in the study, all subjects understood the content of the study and signed the informed consent. All participants will fill in a self-designed questionnaire, including general demographic information, lifestyle risk factors such as smoking and drinking, physical indicators measurement, the history of major diseases and other data. Smokers are those who report smoking at least once a day for a year or more. Drinking is the sum of milliliters of alcohol extracted weekly from wine, beer and spirits. Hypertension patients were those whose SBP was equal or more than 140 mmHg and/or DBP was equal or more than 90 mmHg and/or use of antihypertensive medication.

### Genomic DNA extraction and genotyping

We selected the SNPs according to two methods as following: firstly, we selected SNPs from *ELMO1* gene family; secondly, we selected the SNPs within *ELMO1* gene, which have been reported in previous studies, but no consistent results were obtained on the relation between SNPs and DN risk. According to the standard instructions, we will take 3 ml blood samples from all participants, which are processed by EDTA and stored in a refrigerator at – 20 °C for DNA genomic DNA extraction. Genotyping was performed with a polymerase chain reaction (PCR) and following restriction fragment length polymorphism (RFLP). The description, primers and enzyme for the four SNPs were shown in Additional file 1: Table S1, including rs741301, rs1345365, rs10255208 and rs7782979. A 25 μl reaction mixture including 1.25 μl SNP Genotyping Assays (20×), 12.5 μl Genotyping Master Mix (2×), 20 ng DNA. PCR cycling conditions consisted of an initial denaturation at 96 °C for 7 min, followed by 35 cycles of 96 °C for 20 s, 57 °C for 30 s, and 72 °C for 45 s, ending with a final elongation step at 72 °C for 5 min. For quality control, genotyping was performed with blinding to the grouping of participants, and both controls and cases were randomly selected 10%, which were genotyped for two times by different staffs, and the reproducibility should be 100%.

### Statistical analysis

In current study, the Hardy–Weinberg equilibrium (HWE) test and comparison of distribution of alleles and genotypes between case and control groups were performed with Chi-squared test. The mean ± standard deviation (SDS) was used to represent the continuous variables of normal distribution, and Student t test was used to compare the case group and the control group. Logistic regression model was used to calculate the statistical relationship between four SNPs and DN risk. The best interaction combinations associated with DN among the 4 SNPs in *ELMO1* gene was determined with generalized multifactor dimensionality reduction (GMDR), a sign or permutation test (providing empirical *p* values) used for predicting accuracy was employed to measure the significance of an identified model. Statistically significance was determined when *p*-values was less than 0.05.

## Results

A description for DN patients and normal controls regarding demographic and general or clinical characteristics is shown in Table 1. A total of 1325 participants including 660 T2DM patients with DN and 665 T2DM patients without DN (controls). The average age for all participants was 66.0 ± 12.8 years. There was no significant difference found in several parameters, including gender, smoking rates, age, duration of diabetes, FPG and BMI between the two groups (all *p*-values were more than 0.05). In contrast, the percentages of participants who consumed alcohol, hypertension patients, means of HbA1c, creatinine, urea and urine albumin/creatinine ratios (ACR) were higher in the DN patients than that in controls.

**Table 1:** General characteristics of 1325 study participants in case and control group

Variables	DN patients (n = 660)	Normal controls (n = 665)	*p*-values
Age (year), mean ± SD	65.8 ± 13.8	66.3 ± 14.3	0.517
Gender, N (%)			0.652
Males, N (%)	378 (57.3)	389 (58.5)	
Females, N (%)	282 (42.7)	276 (41.5)	
Hypertension, N (%)	238 (36.1)	195 (29.3)	0.009
BMI (kg/m^2^), mean ± SD	24.3 ± 8.4	23.7 ± 8.1	0.186
Smoking, N (%)			0.062
Never smoking	435 (65.9)	470 (70.7)	
Ever or current smoking	225 (34.1)	195 (29.3)	
Alcohol drinking, N (%)			0.0033
Never drinking	408 (61.8)	462 (69.5)	
Ever or current drinking	252 (38.2)	203 (30.5)	
FPG (mmol/l)	9.3 ± 3.9	9.1 ± 3.7	0.338
HbA1c (%)	8.57 ± 3.23	8.28 ± 3.25	0.029
Duration of diabetes	10.1 ± 4.4	9.8 ± 4.7	0.231
Creatinine (mg/dl)	1.24 ± 0.37	1.12 ± 0.29	< 0.001
Urea (mg/dl)	40.5 ± 15.1	35.3 ± 14.3	< 0.001
ACR (μg/mg)	82.34 ± 33.8	22.8 ± 7.6	< 0.001

*BMI* body mass index, *T2DM* type 2 diabetes mellitus, *FPG* fasting plasma glucose, *ACR* urine albumin/creatinine ratio

The genotype frequencies in the control of the current study were all distributed accordingly to HWE (Table 2). The frequency of the rs741301-G allele was 30.7% in DN patients and 20.2% in normal controls, in addition, the frequency of the rs10255208-G allele was 28.3% in DN patients and 20.4% in normal controls, which was also indicating a significantly statistical difference. The rs741301-G allele and the rs10255208-GG genotype were associated with an increased risk of DN risk, adjusted ORs (95% CI) were 1.75 (1.19–2.28) and 1.41 (1.06–1.92), respectively. We also found that the others SNPs-rs1345365 and rs7782979 were not correlated with DN susceptibility significantly.

**Table 2:** Association analysis for four SNPs within ELMO1 gene and DN susceptibility

SNPs	Genotypes or alleles	Frequencies N (%)		OR (95% CI)^a^	*p*-values	*p*-values for HWE test in controls
		Normal controls (n = 665)	DN patients (n = 660)			
rs741301	AA genotype	430 (64.7)	325 (49.2)	1.00 (ref)		0.148
	AG genotype	202 (30.4)	265 (40.2)	1.68 (1.15–2.23)	< 0.001	
	GG genotype	33 (5.0)	70 (10.6)	2.04 (1.29–2.82)	< 0.001	
	A allele	1062 (79.8)	915 (69.3)	1.00 (ref)		
	G allele	268 (20.2)	405 (30.7)	1.75 (1.19–2.28)	< 0.001	
rs1345365	AA genotype	415 (62.4)	382 (57.9)	1.00 (ref)		0.228
	AG genotype	214 (32.2)	235 (35.6)	1.20 (0.80–1.81)	0.462	
	GG genotype	36 (5.4)	43 (6.5)	1.51 (0.69–2.32)	0.613	
	A allele	1044 (78.5)	999 (75.7)	1.00 (ref)		
	G allele	286 (21.5)	321 (24.3)	1.24 (0.77–1.95)	0.529	
rs10255208	AA genotype	426 (64.1)	351 (53.2)	1.00 (ref)		0.294
	AG genotype	207 (31.1)	245 (37.1)	1.28 (0.88–1.77)	0.425	
	GG genotype	32 (4.8)	64 (9.7)	1.90 (1.30–2.59)	< 0.001	
	A allele	1059 (79.6)	947 (71.7)	1.00 (ref)		
	G allele	271 (20.4)	373 (28.3)	1.41 (1.06–1.92)	0.021	
rs7782979	CC genotype	409 (61.5)	376 (57.0)	1.00 (ref)		0.592
	CA genotype	228 (34.3)	242 (36.7)	1.20 (0.84–1.72)	0.487	
	AA genotype	28 (4.2)	42 (6.4)	1.29 (0.76–1.97)	0.641	
	C allele	1046 (78.6)	994 (75.3)	1.00 (ref)		
	A allele	284 (21.4)	326 (24.7)	1.23 (0.82–1.76)	0.583	

^a^Adjusted for age, gender, BMI, hypertension, smoking and alcohol drinking

**Fig. 1:**
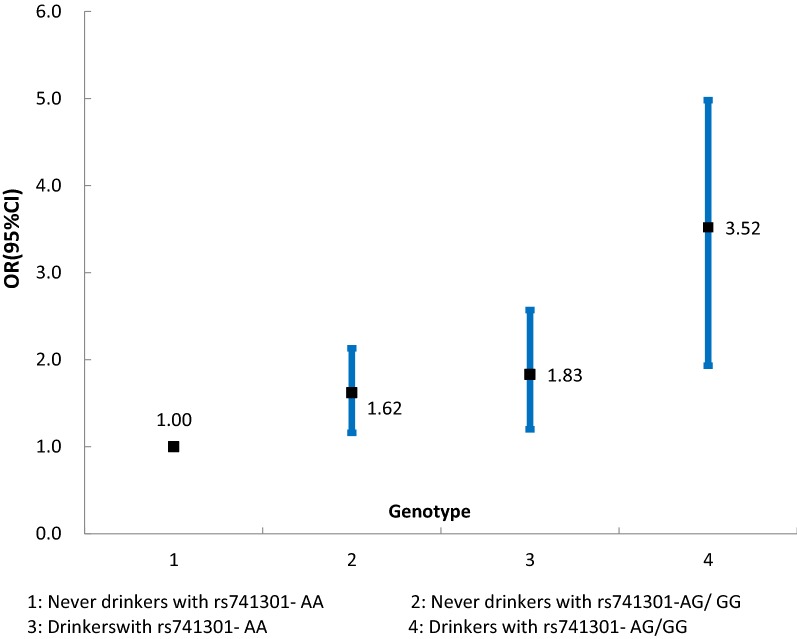
Stratified analysis for rs741301-alcohol drinking interaction on DN risk

The gene–hypertension or alcohol drinking interaction test were determined by GMDR model (Table 3). We found a significant gene–alcohol drinking interaction combination, but no significant gene–hypertension interaction combinations, after adjusting for age, gender, BMI, smoking and alcohol drinking covariates. A two-locus including rs741301 and alcohol drinking was significantly in the GMDR model. In order to obtain the odds ratios and 95% CI for the joint effects of gene–alcohol drinking on DN, we conducted stratified analysis for interaction effect using logistic regression. We found that alcohol drinkers with rs741301-AG/GG genotype also have the highest DN risk, compared to never drinkers with rs741301-AA genotype, OR (95% CI) 3.52 (1.93–4.98) (Fig. 1).

**Table 3:** GMDR analysis for the best interaction combination models

Locus no.	Best combination	Cross-validation consistency	Testing balanced accuracy	p-values*
Gene–hypertension interactions^a^				
2	1, hypertension	8/10	0.598	0.6241
3	1, 3, hypertension	6/10	0.562	0.377
4	1, 2, 3, hypertension	5/10	0.545	0.857
5	1, 2, 3, 4, hypertension	7/10	0.521	0.377
Gene–alcohol drinking interactions^b^				
2	1, alcohol drinking	*10/10*	*0.621*	*0.0107*
3	1, 3, alcohol drinking	8/10	0.599	0.1719
4	1, 2, 4, alcohol drinking	7/10	0.558	0.3770
5	1, 2, 3, 4, alcohol drinking	8/10	0.476	0.5316

SNPs named with 1–4 were rs741301, rs1345365, rs10255208 and rs7782979 respectively

^a^Adjusted for age, gender, BMI, smoking and alcohol drinking

^b^Adjusted for age, gender, BMI, hypertension and smoking

## Discussion

In the current study, we found that rs741301-G allele and the rs10255208-GG genotype were associated with an increased risk of DN risk respectively. We also found that the others SNPs-rs1345365 and rs7782979 were not significantly associated with susceptibility to DN. DN is the most common cause of ESRD and the most common microvascular complication of diabetes mellitus [17]. However, its detailed pathogenesis was not well known yet, and both environmental and genetic factors were reported involving in the occurrence and development of DN susceptibility [18, 19]. *ELMO1* is a protein composed of 720 amino acids, encoded by elmo1 gene and located on chromosome 7p14.2-14.1. It may be a new and powerful candidate gene of DN, which has influence on the movement and phagocytosis of apoptotic cells [20]. Animal studies in mice [21] suggest that *ELMO1* protein plays an important role in the pathogenesis of DN and proteinuria. Over the last decade, some studies were performed to test the relationship between *ELMO1* gene SNPs and DN risk, but these studies concluded inconsistent results, and few studies were performed in Chinese populations. Mehrabzadeh et al. [22] investigated the relationship between *ELMO1*-rs741301 and the risk of DN in Iranian population, and considered that *ELMO1*-rs741301 is an important candidate gene for DN susceptibility. Bodhini et al. [23] reported a significant association of the *ELMO1* rs741301 SNPs with DN in south Indians. Wu et al. [11] also verified the association of rs741301 with DN, and rs1345365 was not associated with DN risk, however the sample size for this study was very small, just 200 unrelated Chinese subjects were enrolled. Some studies including GWAS and replication studies were performed in Japanese type 2 diabetic patients [8], replication studies in American Indian study [10] and GoKinD collection study [9], African American cohorts [24]. The key role of *ELMO1* as a DN susceptibility gene has been verified. Although many studies have confirmed that elmo1 is a candidate gene for DN, the risk locus and risk allele are not consistent in all populations [25]. Different perspectives also existed in some studies. Yahya et al. [26] suggested that *ELMO1*-rs74130 was not associated with susceptibility to DN. Hanson et al. [10] indicated that *ELMO1*-rs1345365, but not rs741301 associated with DN risk. In terms of rs10255208, which was less studies previously, but this SNP has been reported association with T2DM in Tunisian Arabs [27].

The pathogenesis and process of DN are very complex. The factors of DN susceptibility mainly include genetic factors, environmental factors and the synergistic effect of genetic and environmental factors [28, 29]. Some studies have showed that the interaction between gene and environment factors were associated with the DN risk. This study is the first to confirm the synergistic effect of elmo1 gene polymorphism and environmental factors on DN susceptibility. We found a significant gene–alcohol drinking interaction combination, but no significant gene–hypertension interaction combinations. Alcohol drinkers with rs741301-AG/GG genotype also have the highest DN risk, compared to never drinkers with rs741301-AA genotype. The detailed mechanism for *ELMO1* gene-DN association was not well known. However, a previous study [12] showed that mutations in elmo1 gene can lead to the disorder of ECM metabolism, the accumulation of which could thicken of renal tubules and glomerular basement membrane, thus increasing the risk of DN. Another study [21] suggested that *ELMO1* also could plays an important role in the development of DN by increasing oxidative stress (OS), the level of elmo1 gene expression in diabetic mice was consistent with the degree of renal fibrosis and urinary albumin excretion. There was also a positive correlation between ROS and *ELMO1* expression.

The limitations of this study were: Firstly, more environment factors should be included in the interaction analysis; secondly, the study population were all Chinese Han, which may not present on behalf of the Chinese populations, because there were 56 races in China. Secondly, just four SNPs were selected for genotyping, more SNPs should be included, and gene–gene interaction should be investigated in the future.

In conclusion, we found that the rs741301-G allele and the rs10255208-GG genotype, gene–environment interaction between rs741301 and alcohol drinking were all associated with increased DN risk. This study not only investigated whether the *ELMO1* gene was related to DN susceptibility, but also investigated whether the interaction effect existed between this gene and some significant environmental factors, such as hypertension, alcohol drinking. Previous studies merely investigated this interaction effect between *ELMO1* gene and alcohol drinking, especially in Chinese Han populations.

## Supplementary information

**Additional file 1: Table S1.** Description and primer sequences designed for sequencing 4 SNPs within ELMO1 gene.

## Data Availability

Not applicable.
