# Supplementary material for: Interaction between ELMO1 gene polymorphisms and environment factors on susceptibility to diabetic nephropathy in Chinese Han population
Source: Diabetol Metab Syndr. 2019 Nov 27;11:97. doi: 10.1186/s13098-019-0492-0 (PMC6882154; doi:10.1186/s13098-019-0492-0)
Supplement: Supplementary file 1 — Additional file 1: Table S1. Description and primer sequences designed for sequencing 4 SNPs within ELMO1 gene. [file 13098_2019_492_MOESM1_ESM.docx]

| Table S1. Description and primer sequences designed for sequencing 4 SNPs within ELMO1 gene | | | | | |
| --- | --- | --- | --- | --- | --- |
| SNPs | Chromosome | Functional Consequence | Major/ minor alleles | Enzyme | Primer (5’→3’) |
| rs1345365 | 7:37161008 | Intron variant | A/ G | *EcoP15I* | Forward: 5′- GCCACTTCTTCCCCTACAACATTGA -3′  Reverse:5′-GCCAGTGAGAGAGTAATACTATTACGTTC-3′ |
| rs741301 | 7:36878390 | Intron variant | A/ G | *TsoI* | Forward: 5′-ACGTTGGATGCAGTTCCCATGGTGGTTATC -3′  Reverse:5′-ACGTTGGATGGAACTCTTCAAGCTCAATAG -3′ |
| rs10255208 | 7:36819038 | - | A/ G | *Tsp45 I* | Forward: 5′- AAGTTGGACCCCTGCCTCACA -3′  Reverse: 5′- GCCCCTGAGCTGACCGTTCT -3′ |
| rs7782979 | 7:36865445 | Intron variant | C / A | *Mse I* | Forward: 5′- AACTCGGCATGTGTGTGGGTG -3′  Reverse: 5′- AGGAATTGGCCAGGCAGAGA -3′ |
